# An analysis of environment effect on ethanol blends with plastic fuel and blend optimization using a full factorial design

**DOI:** 10.1038/s41598-022-26046-9

**Published:** 2022-12-15

**Authors:** S. Padmanabhan, T. Vinod Kumar, K. Giridharan, B. Stalin, N. Nagaprasad, Leta Tesfaye Jule, Krishnaraj Ramaswamy

**Affiliations:** 1 School of Mechanical and Construction, Vel Tech Rangarajan Dr.Sagunthala R&D Institute of Science and Technology, Chennai, India; 2 Department of Mechanical Engineering, Vels Institute of Science, Technology and Advanced Studies (VISTAS), Chennai, India; 3 Department of Mechanical Engineering, Easwari Engineering College, Chennai, India; 4 Department of Mechanical Engineering, Anna University, Regional Campus Madurai, Madurai, Tamilnadu 625019 India; 5 Department of Mechanical Engineering, ULTRA College of Engineering and Technology, Madurai, Tamilnadu 625104 India; 6 Centre for Excellence-Indigenous Knowledge, Innovative Technology Transfer and Entrepreneurship, Dambi Dollo University, Dambi Dollo, Ethiopia; 7 Department of Physics, College of Natural and Computational Science, Dambi Dollo University, Dambi Dollo, Ethiopia; 8 Department of Mechanical Engineering, Dambi Dollo University, Dambi Dollo, Ethiopia

**Keywords:** Environmental sciences, Chemistry, Energy science and technology, Engineering, Materials science

## Abstract

There is a growing amount of plastic waste that needs to be properly disposed of in order to protect the environment from the negative effects of increasing reliance on plastic products. Recent interest has focused on chemical recycling as a means of reducing plastic's negative environmental effects. Converting waste plastics into basic petrochemicals allows them to serve as hydrocarbon feedstock or fuel oil through pyrolysis operations. Scientists have taken a keen interest in the production of bioethanol from renewable feedstocks due to its potential as a source of energy and alternative fuel. Due to its beneficial effects on the environment, ethanol has emerged as a promising biofuel. In this paper, energy recovered from low-density polyethylene and high-density polyethylene waste was converted into an alternative plastic fuel and evaluated for its environmental impact with the blending of ethanol in a diesel engine. Ternary fuel blends with 20%, 30%, and 40% waste plastic fuel and 10%, 15%, and 20% ethanol with standard diesel were tested. The study found that blending 10% ethanol with 20% plastic fuel decreased fuel consumption by around 7.9% compared to base diesel. Carbon monoxide emissions are reduced by about 10.2%, and hydrocarbon emissions are reduced by about 13.43% when using the same ternary blend. The optimum values of fuel consumption and emissions were obtained by full factorial design for a ternary fuel blend of 10% ethanol and 20% plastic fuel at the full load condition.

## Introduction

Since the invention of the first synthetic plastic, both its production and use around the world have skyrocketed. The problem is that plastics last a long time and do not break down easily, so they end up as waste that sticks around for a while. Waste management issues, improper recycling, and littering contribute to plastic pollution, which has far-reaching negative effects on the natural world. Reusing and recycling only account for a small percentage of plastic trash at this time. Plastic recycling has yet to reach its full potential due to technical difficulties and low revenues, especially for mixed plastics^1^. The average lifespan of a plastic product is only one day to three years. However, the time required for these goods to decompose is still excessively long; for instance, it takes plastic products an average of 300–500 years to deteriorate^2^. Many plastic pieces are contaminated with metals, paper, other kinds of polymers, and/or other fillers, preventing mechanical recycling of the entire plastic collection. Approximately 70% of plastic garbage is burnt or disposed of in landfills around the world. Landfilling and incineration with minimal energy recovery are not viable long-term solutions for plastics because they are derived from crude oil and natural gas products. For polluted and mixed chemicals, recycling is a viable option^3^. In addition to solar and wind power, the burning of a plastic garbage can be used to recover energy. It is possible that these hydrocarbon polymers can be used to replace fossil fuels, hence reducing emissions of carbon dioxide. This is because the energy produced by incinerating polyethylene is about equivalent to that used in its production and because polyethylene has a calorific value similar to that of fuel oil^4^. Recycling plastics is a good way to reduce plastic pollution. Mechanical reprocessing is the primary technique of plastic recycling; however, it only works with high-purity, selectively selected garbage. Thermal or catalytic recycling creates hydrocarbon, which is appealing. Compared to other procedures, pyrolysis is cost-effective. Pyrolysis thermally destroys the plastic component to create oil and gas^5^.

Plastic oil is the byproduct of processing polyethylene, polypropylene, and polystyrene. During the experiments, researchers manipulate fuel injection time by adding shims to the engine, which retards timing, and removing shims, which advances the timing. Timing fuel injection enhances performance and minimizes emissions. By modifying fuel injection timing in advance, they reduce Break fuel usage, Nitrogen Oxides (NOx) emissions, and smoke emissions. Retard's fuel injection timing has been lowered to reduce NOx emissions, improving engine performance^6^. Waste plastic fuels produce more NOx than diesel. Due to the engine's inability to properly ignite the pyrolysis fuels at the outset of combustion, carbon emissions increase. The smoke index is decreased via carbon-chain-link length reduction. But the brake thermal efficiency and fuel consumption were benefited from higher heating value and cetane index^7^.

Ethanol's volumetric efficiency is enhanced by its many positive qualities, including its low viscosity, high oxygen content, high hydrogen/carbon ratio, low sulphur content, and strong evaporative cooling. However, diesel engines may utilize ethanol when combined with diesel fuel. Ethanol's reduced viscosity compared to diesel enables improved atomization of the fuel supplied into the cylinders and enhanced mixing with air. Due to its high latent heat of evaporation, ethanol can be used to improve volume efficiency in a diesel engine when blended with biodiesel fuel. This is accomplished through evaporative cooling of ethanol during engine operations^8^. Typically, ethanol can make up to 20% of the total volume of diesel fuel when combined with diesel. The blend's stability, cetane number, and lubricity are all affected by the substance employed to stabilize it. Increased alcohol content improves engine performance, reduces emissions, and lowers density and kinematic viscosity^9^.

In all engine loads, Waste Plastic Oil (WPO) burns similarly to diesel fuel; however, castor oil slows combustion compared to palm biodiesel. Both biodiesel blends were shown to increase efficiency and decrease smoke emissions in comparison to diesel. The WPO in biodiesel helped cut down on harmful emissions of hydrocarbons and nitrogen oxides. Both carbon monoxide and smoke emissions rose when biodiesel was blended with WPO^10^. When the n-butanol concentration in the blends was increased, a researcher observed improved thermal efficiency. A 10% butanol blend in plastic fuel and diesel was found to decrease NOx and smoke pollutants while increasing engine performance^11^. According to the tests conducted, the most effective mixture was one containing 50% diesel, 30% waste plastic oil, and 20% n-hexanol by volume. At full load, this mixture improves brake thermal efficiency by about 1.3% and 14% compared to pure diesel and waste plastic oil while also reducing oxides of nitrogen pollution by about 4% and reducing smoke emission by about 30% and 40%, respectively^12^.

The ethoxy ethyl acetate and ethanol as oxygenated additives were blended with fuel produced from the high-density polyethylene by the pyrolysis process. Due to the high oxygen content, low viscosity, and high carbon/hydrogen ratio of ethanol, quaternary fuels offer greater thermal efficiency, reduced fuel usage, and reduced harmful emissions^13^. Ethanol is a renewable biofuel that serves as an oxygenated fuel in diesel engines, reducing emissions. Three percentages of polyethylene plastic pyrolysis oil were blended with ethanol to form ternary fuel. W20E10's fuel usage drops 7.5% with diesel. W20E10 had lower carbon monoxide emissions than diesel and hydrocarbon at varied loads^14^.

Increasing the load and compression ratio, as well as adding waste plastic oil and ethanol to diesel, boosts thermal efficiency and emission quality. Using ANOVA and multivariate analysis, the appropriate engine load, compression ratio, and fuel blend were identified, improving performance and reducing emissions. Maximum braking thermal efficiency and reduced emissions were observed with a compression ratio of 18.1, and 20% WPO and 20% ethanol blended diesel^15^. As opposed to full factorial designs, the calibration procedure can be completed in a fraction of the time using DoE approaches for the reduction of experimental plans to determine the optimal values for the injection parameters. It was also mentioned that the optimization process might be heavily impacted by how the goal function is formulated^16^. In order to reduce NOx and smoke emissions while maintaining optimal fuel efficiency, a response surface methodology-based optimization using a three-factor, a three-level full factorial experimental design was implemented. Statistically, significant multiple surface regression models were found for NOx, smoke density, and fuel consumption. Injection timing and EGR were compared for all fuel blends^11^.

It was found in the literature that a variety of pyrolysis reactors were utilized to convert individual plastics into liquid products^17^. But there is not much data on how to turn various types of plastics into useful recovery. In this paper, energy recovered from low-density polyethylene (LDPE) and high-density polyethylene (HDPE) waste into an alternative plastic fuel was evaluated for its environmental impact with the blending of ethanol in a diesel engine. Ternary fuel blends with 20%, 30%, and 40% waste plastic fuel and 10%, 15%, and 20% ethanol with standard diesel were tested. The performance and emission characteristics of a diesel engine are studied and assessed. The fuel consumption and emissions were studied through a full factorial design on the influence of engine load, ethanol, and plastic fuel blend ratios.

## Material and methods

Plastics have proved to be "one of the greatest innovations of the millennium." Plastic is lightweight, does not rust or rot, is cheap, reusable, and saves natural resources; hence it is popular^18,19^. India produces 10 million tons of plastic annually. Three million metric tons of plastic waste are dumped daily, but 70% is recycled. Most nations recycle and burn plastic waste to reduce its impact. Pyrolysis is the most efficient way to recycle plastic since the energy may be used directly. Pyrolysis reduces waste volume by 90%, produces solid, liquid, and gaseous fuels, and has the least environmental impact of any thermal waste degradation process^20^.

Researchers studied the influence of polystyrene (PS), polyethylene (PE), polypropylene (PP), and polyethylene terephthalate (PET) on pyrolysis oil yield and quality. PS plastic trash produced the most liquid oil (80.8%) and the least fumes (13%), char (6.2%). All liquid plastics contain aromatic chemicals, alkanes, and alkenes^21^. Hydrocracking at 350 °C with 20 mL/min of hydrogen gas stream took one hour. LDPE plastic waste converted over Co-Mo/Z at 350 °C yielded 71.49% fuel selectivity. Catalysts lower liquid and raise gaseous fractions. Based on GCMS data, liquid yield comprised of C6-C19 hydrocarbon compounds, such as paraffin, olefins, and naphthenes^22^. Commercial and natural Y-zeolite catalysts were studied. The feedstock types affect liquid and solid product yields and quality. HDPE waste produces more fuel yield^23^.

Table 1 lists the characteristics of the various plastics like polyethylene, polypropylene, polyethylene terephthalate, and polyvinyl chloride found in plastic garbage. Shredding the plastic into pieces between 2 and 5 mm was a necessary step in the recycling process. All of the plastics were broken down using the catalytic pyrolysis technique, and their individual characteristics were analyzed and compared^25^. The pyrolysis process is used to recover energy, with liquid fuel as the major product. The pyrolysis procedure was carried out using a reactor-furnace combination equipped with a PID controller. The reactor was filled with a ZSM (zeolite) to plastics, and the ratio was a 1:4 combination. The reactor was heated from 450 to 600 °C in a vacuum-sealed furnace. A container was placed at the base of the reactor in order to collect the condensed liquid^26,27^.

**Table 1:** Properties of plastic materials^24^.

Type of Plastics	Fixed carbon (wt%)	Ash (wt%)	Moisture (wt %)	Volatile (wt%)
Polyethylene (PE)	0.04	0.99	0.10	98.87
High-density Polyethylene (HDPE)	0.01 0.03	0.18 1.40	0.00 0.00	99.81 98.57
Low-density Polyethylene (LDPE)	0.00 –	0.00 0.40	0.30 –	99.70 99.60
Polypropylene (PP)	1.22 0.16	3.55 1.99	0.15 0.18	95.08 97.85
Polyethylene terephthalate (PET)	7.77 13.17	0.02 0.00	0.46 0.61	91.75 86.83
Polyvinyl chloride (PVC)	6.30 5.19	0.00 0.00	0.80 0.74	93.70 94.82

The results of an exhaustive study suggest that waste plastic fuel could be used in ternary fuel blends with oxygenated ethanol, either with or without a modification to the engine settings. According to the findings of the observations, a number of different attempts are currently being undertaken in the laboratory to use alcohols (Table 2) as oxygenated additions in order to improve the performance characteristics and reduce harmful emissions.

**Table 2:**
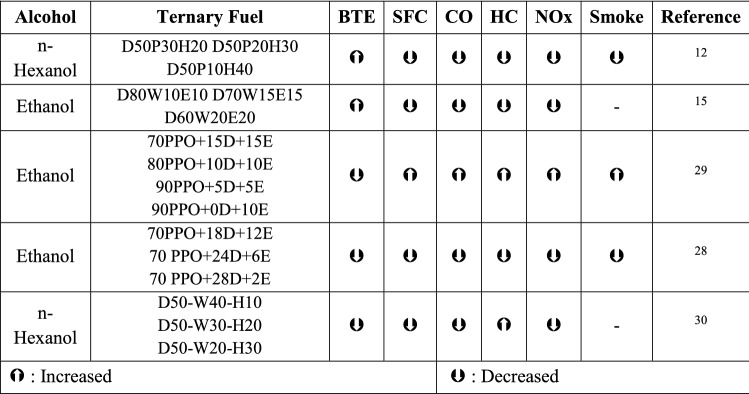
Engine characteristics of alcohols with plastic fuel blends.

Plastic pyrolysis fuel shares many of the same features as diesel fuel, making it a viable alternative fuel source^28,29^. From the above literature, it is possible to recover energy through the pyrolysis process of converting waste plastics to liquid fuel. In this investigation, a unique combination of the shredded HDPE and LDPE was taken in the ratio of 50:50 as the source of plastic oil extraction as plastic fuel (LHP)^30^.

This study looked into the performance and emissions of diesel engines with direct injection, with an investigation of the possibility of using plastic fuel (LHP) extracted from HDPE and LDPE waste and ethanol as a ternary blend. The testing ternary fuel blends were made on a volumetric ratio with standard diesel, plastic fuel (LHP), and ethanol. E10LHP20 ternary blend was created with 70% of diesel, 20% of LHP, and 10% of ethanol. For that, the E15LHP30 blend was made by blending 55% of diesel with 30% of LHP and 15% of ethanol. The E20LHP40 blend was made by blending 40% of diesel with 40% of LHP and 20% of ethanol. The physicochemical properties of ternary blends are listed in Table 3.

**Table 3:** Comparison of Physico-chemical properties of ternary blends.

Fuel properties	Units	Diesel	Ethanol	LHP	E10LHP20	E15LHP30	E20LHP40
Calorific valve	kJ/kg	45,000	25,000	42,127	39,360	38,605	38,090
Density at 20 °C	g/m^3^	0.834	0.787	0.794	0.758	0.759	0.771
Kinematic viscosity at 40 °C	cSt	2.48	0.78	4.89	2.58	2.78	2.98
Cetane number	–	52	10	68	43	46	47
Oxygen content	wt. %	0	36	4.64	3.71	5.77	8.41

## Experimental setup

In this experiment, a single-cylinder diesel engine with direct injection, constant speed, and water cooling was used to make 4.4 kW of power. Computer-controlled eddy current dynamometer connected with the engine and engine torque. Eddy current dynamometer controller is used to manage engine speed and load by altering dynamometer excitation current. A piezoelectric pressure transducer fitted in the engine cylinder head recorded combustion pressure. Using an accurate crank angle encoder, the crank angle and piston locations were determined. Thermocouples are used to measure the temperature of the coolant, the exhaust, and the air intake. The emission characteristics and smoke intensity were measured using an AVL exhaust di-gas analyzer and an AVL smoke meter, respectively.

**Figure 1:**
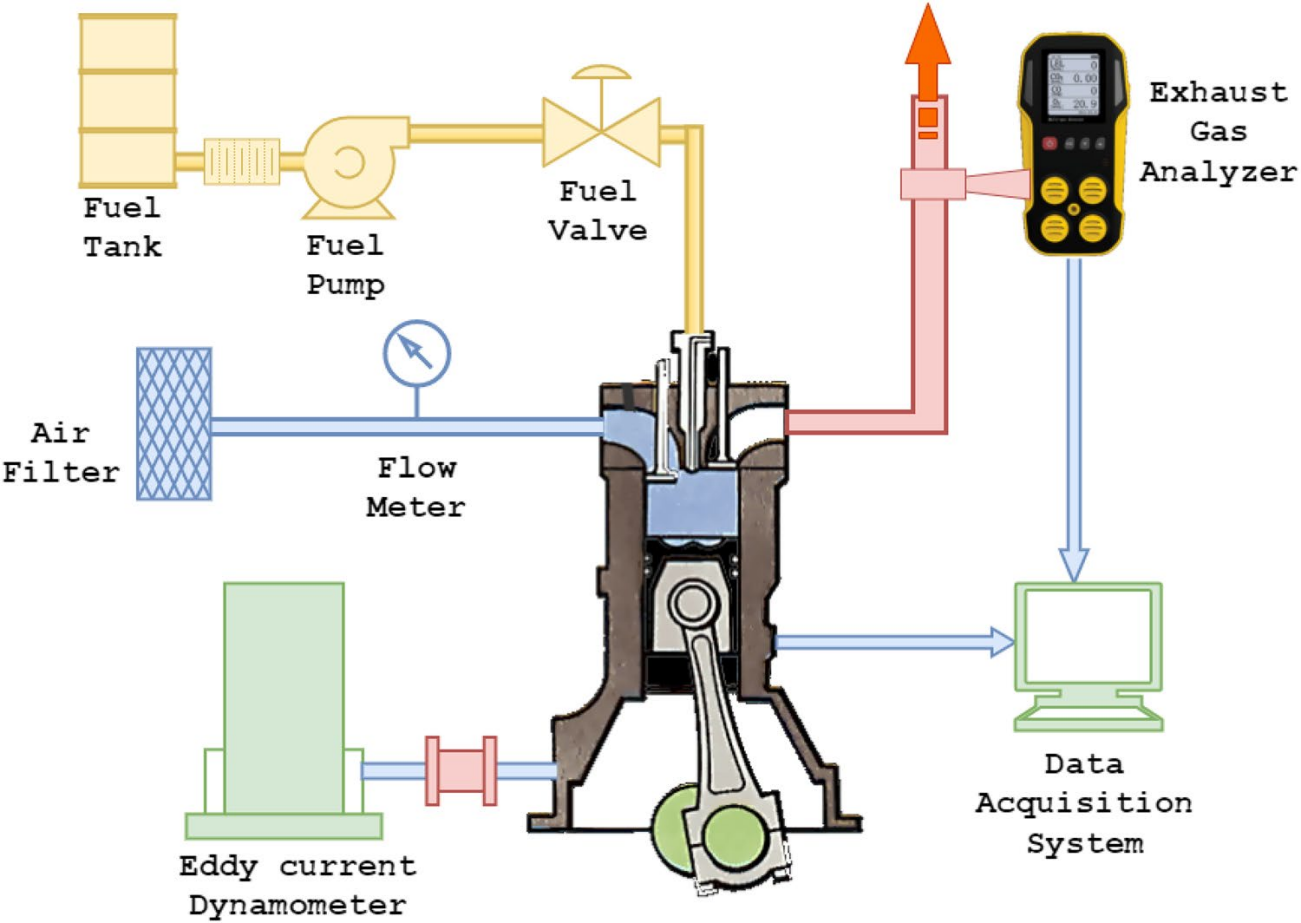
Schematic diagram of experimental engine setup.

The preliminary investigation required starting the engine on diesel and letting it idle for 15 min before subjecting it to any load. Before starting the engine, the air intake, oil level, and fuel level are all inspected. For the engine to be started, both the fuel cutoff lever and the decompression lever had to be in the "on" position while the cranking motion was performed. The decompression lever was released at engine start, and the speed was increased to 1500 rpm and held constant. The investigation was conducted when the engine was running at a steady state speed with a compression ratio of 18:1. The schematic diagram of the experimental engine setup is represented in Fig. 1, and the test engine specification is tabulated in Table 4.

**Table 4:** Experimental test engine specifications.

Parameter	Values
Engine make & type	Kirloskar Engine & Four Stroke Single Cylinder
Maximum power	4.4 kW @ 1500 rpm
Compression ratio	18 : 1
Bore × stroke	87.5 × 110 mm
Injection timing	23° bTDC
Load Type	Eddy current dynamometer

The feasibility of using LHP in a diesel engine by combining ethanol was studied by analyzing engine performance, combustion, and exhaust characteristics. All of the trial test runs were conducted with 100% diesel fuel and kept as base data for comparison. A single-cylinder diesel engine was tested, with engine power increasing by 1.1 kW up to 4.4 kW. The engine was then made to keep running on three ternary test samples, and readings were taken. The brake thermal efficiency, specific fuel consumption, exhaust gas temperature, and combustion parameters like heat release rate and in-cylinder pressure were examined. Among the exhaust gases, carbon monoxide, hydrocarbons, nitrogen oxides, and smoke were recorded.

Experiment uncertainty may result from instrument selection, maintenance, observation, environmental factors, calibration, test design, and reading. Analysis of uncertainty is desired to show how accurate the experiments were. Depending on operational and environmental conditions, performance and measurement accuracy might change^31^. As a result, random or fixed errors are the cause of the uncertainty. Table 5 lists the experimentation critical instrumental parameters and their uncertainty.

**Table 5:** Experimentation critical instrumental parameters and its uncertainty.

Measurement	Range	Accuracy	Percentage of uncertainty	Instrument
Speed	0–10,000 rpm	± 10 rpm	± 0.10	Digital tachometer
Load	–	+ 0.1 kg to − 0.1 kg	± 0.50	Load cell
Fuel quantity	0–50 cm^3^	± 0.1 cm^3^	± 0.50	Burette measurement
Exhaust Temperature	0–1300 °C	± 1 °C	± 0.15	Thermocouple
Carbon monoxide	0–15%	± 0.05%	± 0.15	AVL DI Gas Analyser
Hydrocarbon	0–20,000 ppm	± 10 ppm	± 0.25	AVL DI Gas Analyser
Nitrogen oxides	0–5000 ppm	± 10 ppm	± 0.25	AVL DI Gas Analyser
Smoke	0–100%	± 1%	± 1	AVL Smoke Meter

## Results and discussion

### Study of brake thermal efficiency

**Figure 2:**
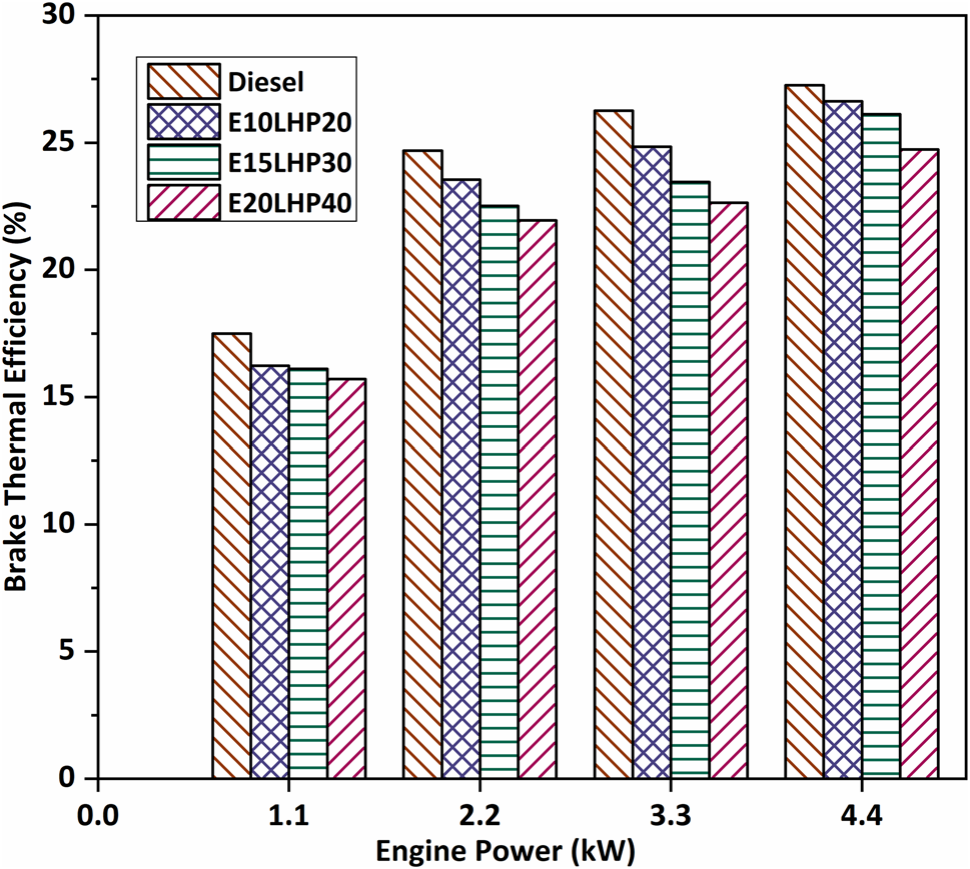
Impact of brake thermal efficiency on LHP and ethanol blends.

Figure 2 shows the calculated brake thermal efficiency (BTE) for the tested diesel engine performance with a ternary fuel of LHP and ethanol. The efficiency was recorded at 5.4%, 10.7%, and 13.8% reduction of BTE for the E10LHP20, E15LHP30, and E20LHP40 blends compared with base diesel. This could be because LHP blends have fewer calories than diesel. At full load, the temperature and rate of heat release from the exhaust gases of plastic fuel are a little higher than those of diesel^10^. A higher proportion of plastic fuel in the blend results in a lesser brake thermal efficiency than base diesel would, as the higher aromatic compound content of plastic oil slows down combustion. The reason for this is that plastic fuel has a high aromatic component, which makes it harder to break the chain. Its higher viscosity and lower thermal efficiency compared to the base fuel tested may be due to problems with fuel injection and the quality of liquid fuel spray^32^.

### Study of specific fuel consumption

**Figure 3:**
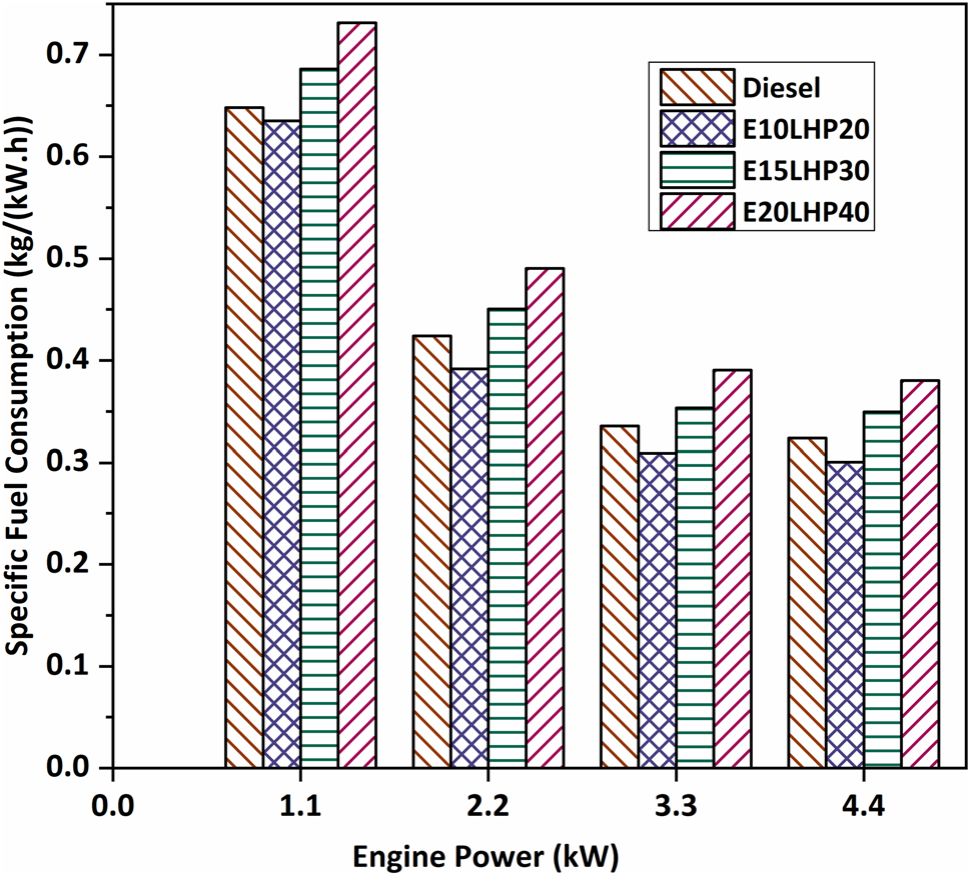
Impact of specific fuel consumption on LHP and ethanol blends.

Figure 3 shows a comparison of the specific fuel consumption (SFC) of three ternary blends with diesel when the various engine load. The specific fuel consumption was reduced by around 2–7.95% when comparing E10LHP20 to diesel as the base fuel at various load levels. Due to better mixing of fuel and air, higher engine speeds lead to better fuel combustion, while higher loads lead to better fuel atomization, better mixing, and a high temperature in the cylinder, all of which help with the combustion process and lead to low specific fuel consumption^33^. As the recorded amount of SFC rises, so does the amount of LHP in the ternary blend. This is because of LHP's increased viscosity and density, which prevents it from being atomized effectively. For E15LHP30 and E20LHP40, the SFC increase from diesel fuel is 7.9% and 17.9%, respectively. The fact that LHP has a lower heating value magnitude and is thicker can explain why it uses more fuel^34^.

### Study of exhaust gas temperature

**Figure 4:**
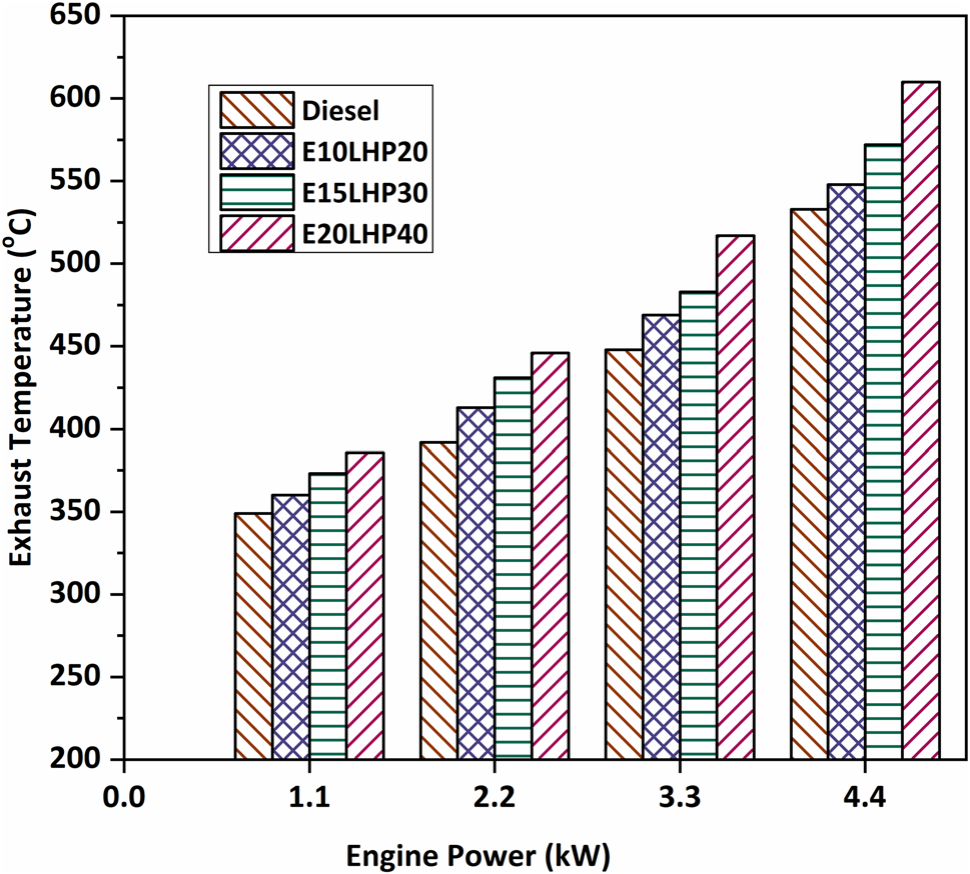
Impact of Exhaust gas temperature on LHP and ethanol blends.

Figure 4 demonstrates that an increase in load causes an increase in the Exhaust Gas Temperature (EGT), which is influenced by the temperature at the combustion process. The exhaust gas temperature recorded at LHP and its blends was found to be higher than base diesel. When LHP is used as a fuel, plastic oil has lower volatility and higher viscosity than conventional fuels, leading to incomplete combustion^35^. As a result, exhaust gas temperature may have increased. EGT was recorded at more than 5.36%, 9.95%, and 15.4% of the base diesel for blends E10LHP20, E15LHP30, and E20LHP40, respectively. It was established that the increased exhaust temperature was brought on by a small quantity of gases traveling through the combustion process at the end of the expansion stroke^15^. Since ethanol contains more oxygen (about 36% by weight), it burns at a higher temperature than diesel. For this reason, LHP blends produce hotter exhaust than expected^32^.

### Study of carbon monoxide emissions

**Figure 5:**
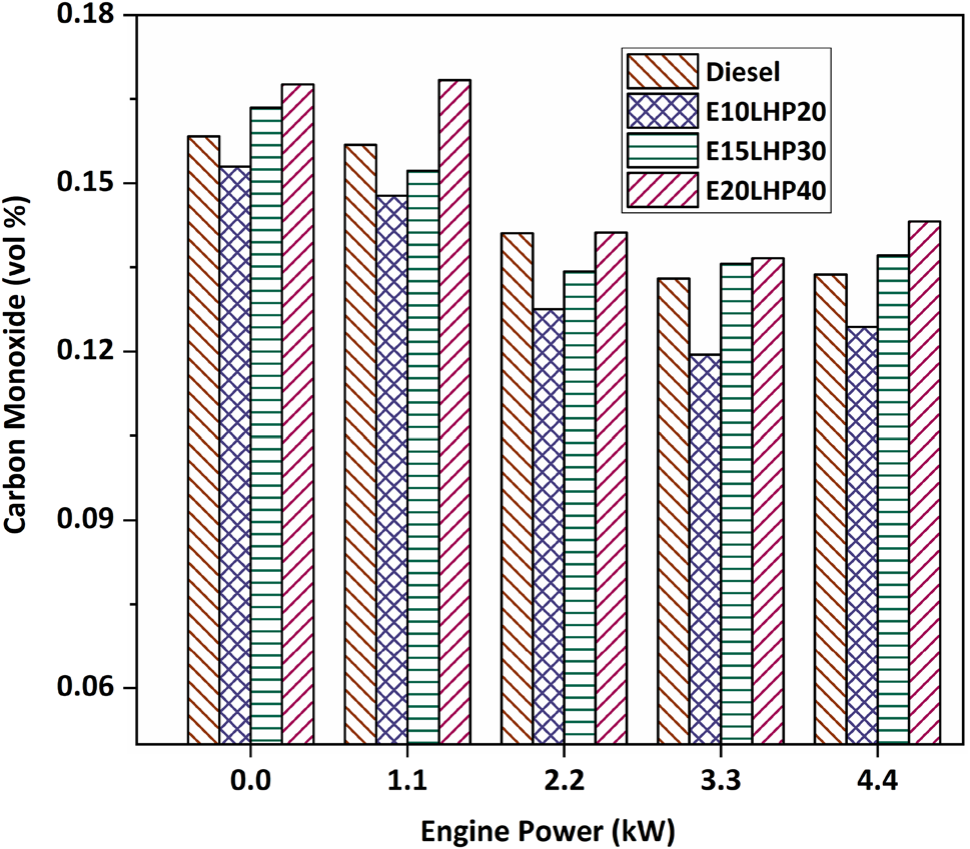
Impact of Carbon monoxide emissions on LHP and ethanol blends.

In the investigation of the E10LHP20 blend under various engine loads, carbon monoxide (CO) emissions were reduced by 3.4–10.2% when compared with base diesel. An LHP blend with higher concentrations of oxygen-rich ethanol burns cleaner and produces fewer carbon dioxide emissions than its binary counterparts^13^. Appropriate combustion led to lower CO emissions when the load was lower, but as the load was increased, combustion efficiency deteriorated, and CO emissions rose for other LHP blends^10^. Figure 5 shows that ternary E15LHP30 and E20LHP40 blends produce significantly more carbon monoxide than diesel. The principal reason for the increase in CO concentration is the increased fuel consumption associated with the increased loads. The increased CO emissions have also been linked to the low in-cylinder temperature seen during combustion. The E15LHP30 and E20LHP40 blends produced a maximum of 3.2% and 7.3% more carbon monoxide than base diesel. The increased viscosity and density of plastic fuel causes inefficient atomization of fuel blend, which in turn increases CO emissions^32^.

### Study of hydrocarbon emissions

**Figure 6:**
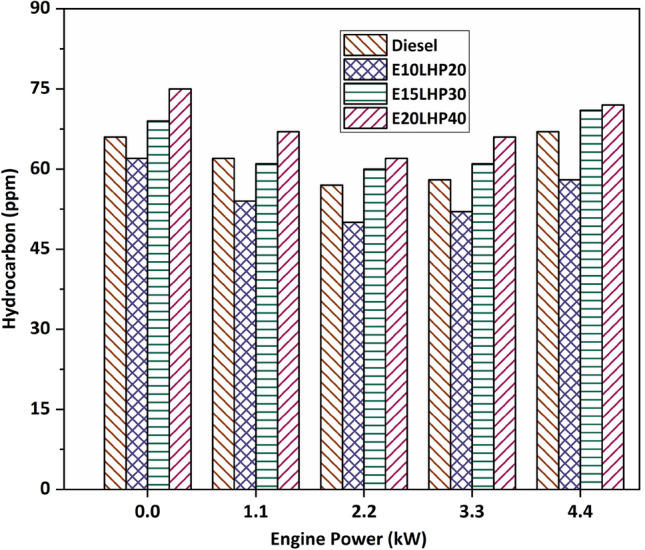
Impact of hydrocarbon emissions on LHP and ethanol blends.

The amount of unburned hydrocarbon (HC) is reduced when the load is lightened because of the higher charge homogeneity and the greater availability of oxygen. When high-oxygen ethanol blends are incorporated into ternary mixtures, combustion efficiency is increased, leading to a decrease in hydrocarbon emissions^36^. The hydrocarbon content of ternary blend E10LHP20 is reduced by around 6–13.43% compared to diesel fuel under varying loading conditions, as shown in Fig. 6. The ternary blends E15LHP30 and E20LHP40 generated a maximum of 5.9% and 13.8% more hydrocarbons than base diesel at various engine powers. The fundamental reason for hydrocarbon emissions is the extinction of the flame in cold parts of the combustion chamber and at the cylinder walls. These emissions are also connected to the viscosity and volatility of the fuel. Larger droplet sizes and lower vapour pressure are two undesirable outcomes of increased viscosity. Unsaturated molecules are responsible for an increase in hydrocarbon emissions, a decrease in auto-ignition, and difficulties in the leaner mixture zone inside cylinder^37^.

### Study of nitrogen oxides emissions

**Figure 7:**
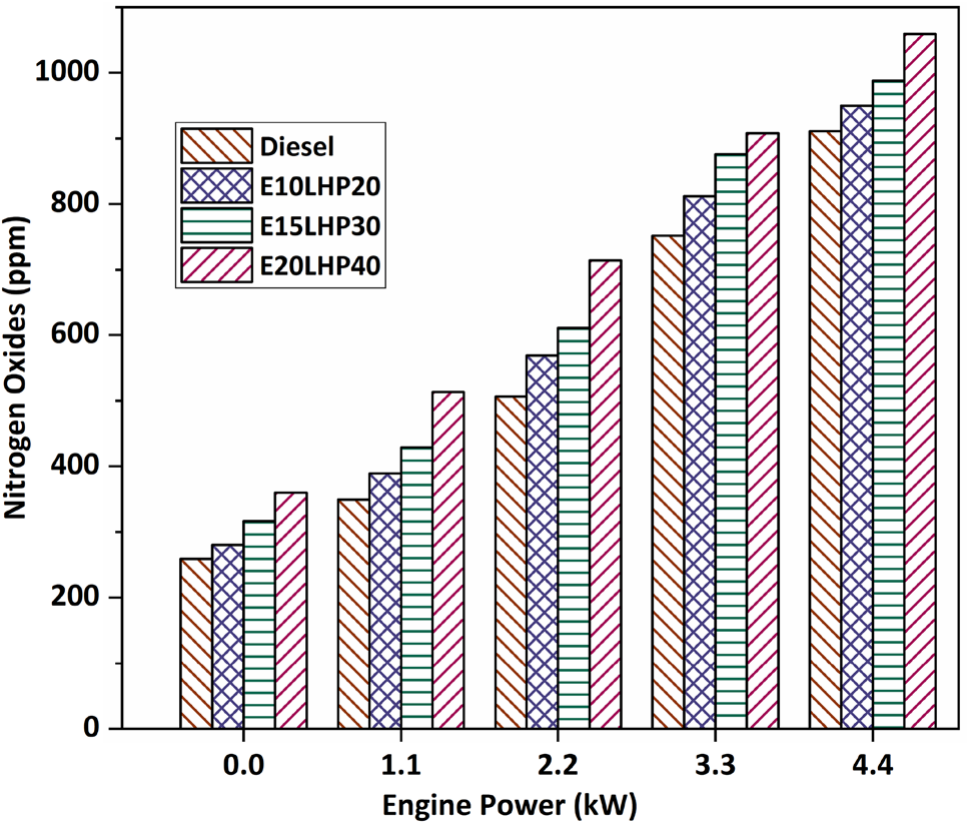
Impact of nitrogen oxides emissions on LHP and ethanol blends.

Figure 7 demonstrates that when the engine load increases, nitrogen oxide emissions from all three ternary fuel blends, E10LHP20, E15LHP30, and E20LHP40, rise relative to those from base diesel. There is speculation that the presence of oxygenated hydrocarbons in plastic fuel contributes to the higher levels of nitrogen oxides in ternary blends compared to diesel. For all of the blends examined, it was shown that NOx emissions increased with increasing load. Massive amounts of nitrogen oxide are generated due to the high temperature and high pressure experienced in the cylinder when employing blends, as well as the emission of massive amounts of heat during combustion^38^. E10LHP20, E15LHP30, and E20LHP40 blends have been recorded as having a maximum of 12.4%, 22%, and 46% more nitrogen oxides than base diesel at various load conditions. This is because ethanol produces more NOx emissions due to its higher oxygen content which is available for burning. The longer time between injecting fuel and starting the engine and the late combustion that results in an increase in NOx^33^.

### Study of Smoke Emissions

**Figure 8:**
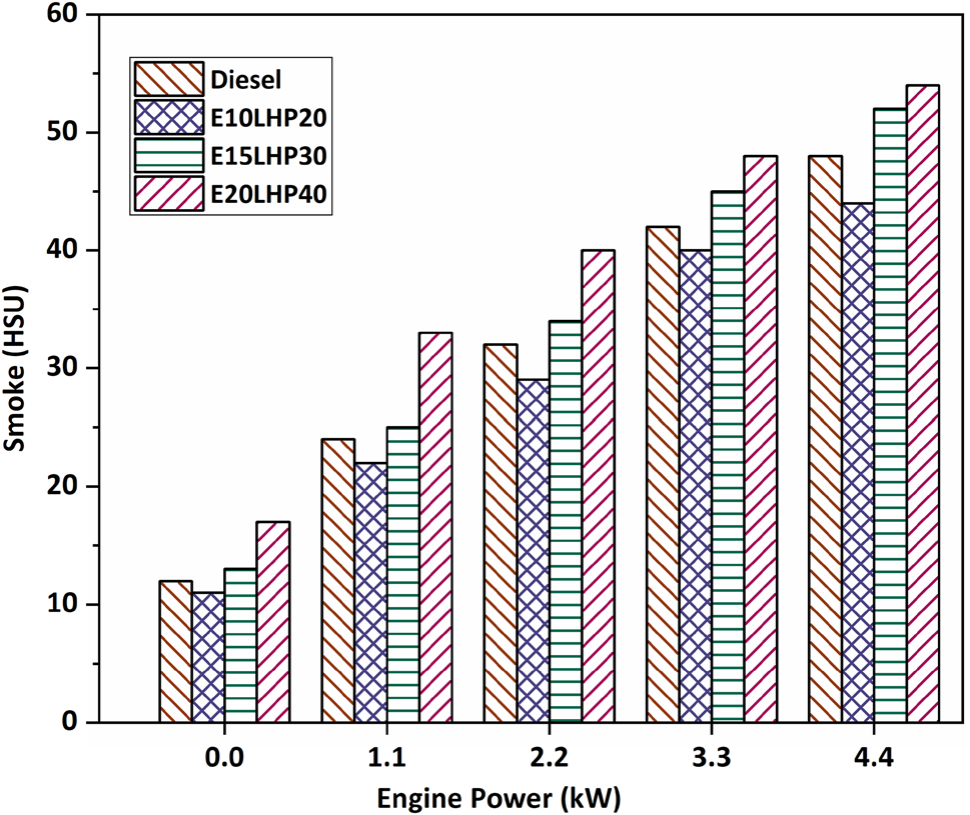
Impact of smoke emissions on LHP and ethanol blends.

In Fig. 8 shows the different test fuel conditions lead to noticeably different smoke patterns. The smoke generated by base diesel is lower than that of E15LHP30 and E20LHP40. The high level of aromatics in LHP leads to an unintended fuel blend. Incomplete combustion and high smoke production have an effect on spray formation. At mid-load, E10LHP20 produces 9.4% less smoke than the base diesel. Simultaneously, E15LHP30 and E20LHP40 emit up to 8.33% and 25% more than diesel, respectively. Causes of smoke include poor atomization, high viscosity, and an excess fuel concentration. Since LHP fuel blends take so long to burn and have such a poor rate of flame spread, they emit a great deal of smoke. Due to increased hydrocarbon combustion, smoke production was exacerbated by incomplete combustion^7,11^.

### Study of in-cylinder pressure

**Figure 9:**
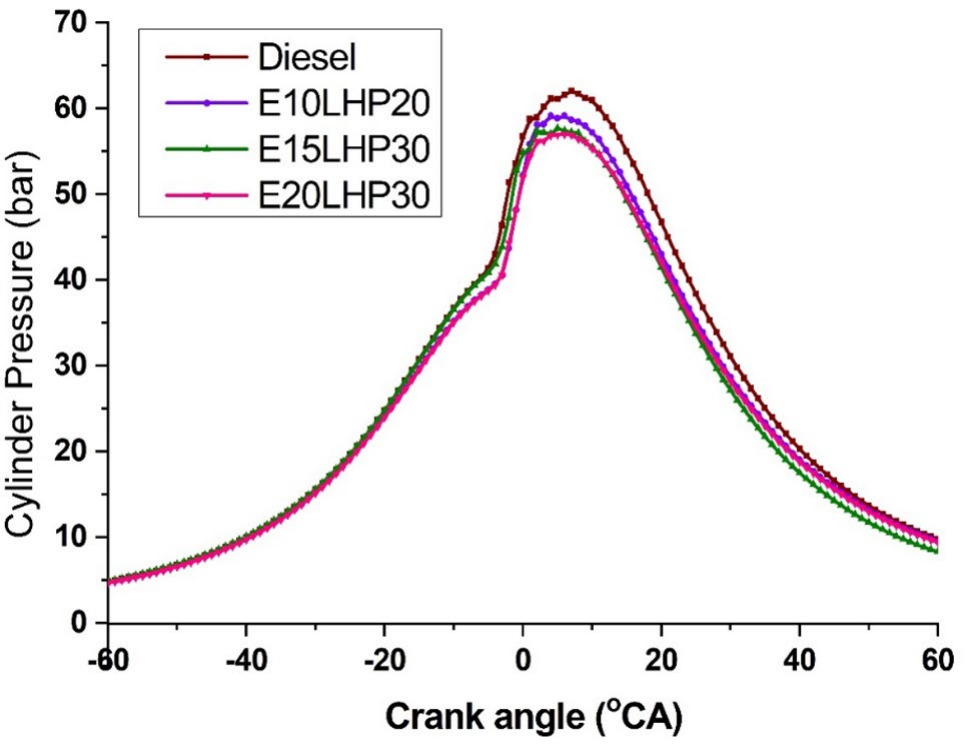
Impact of in-cylinder pressure on LHP and ethanol blends.

The relation between in-cylinder pressure (IP) and crank angle is shown in Fig. 9. For IP measurement, a piezoelectric transducer was used in the combustion chamber. During the first combustion phase, the charge mixer might affect pressure generation. In many cases, the cylinder pressure level is directly related to the charge mixture, fuel viscosity, and cetane number. Engine efficiency and emissions are directly influenced by the combustion parameters, which may be studied extensively through IP analysis. At full load, the highest IP for E10LHP20 was 59.1 bar, whereas the peak IP for diesel was 62.01 bar. Diesel fuels without ethanol had a higher IP than those with ethanol added. This may be because ethanol blends at a cool burning rate and slower oxidation rate than conventional fuels^8^. Diesel pressure generation was also found to be superior in the investigations. This may be attributable to diesel's high energy density and enhanced flammability, which increase evaporation rate and peak cylinder pressure. When ethanol was added to LHP, the IP peak pressure dropped from 59.1 to 56.8 bar. This occurred because ethanol was added, which caused a delay in the ignition and increased the time required for burning. For ethanol associate LHP blends, E10LHP20, E15LHP30, and E20LHP30 all reached peak cylinder pressures of 59.1 bar, 57.3 bar, and 56.9 bar, respectively. The rate of pressure rise is lowered in ethanol-doped blends because of the pooled impact of the lower cylinder temperature and the lower energy content of ethanol, which restrict the generation of high pressure in cylinder^34^.

### Study of heat release rate

**Figure 10:**
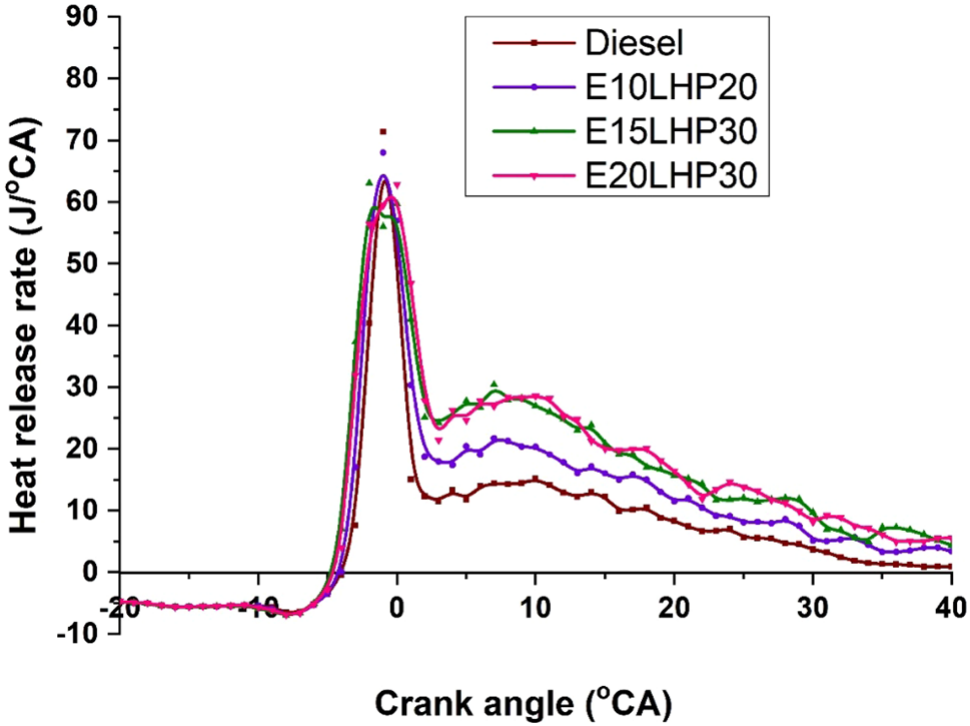
The difference in heat release rate for LHP and ethanol blends.

Figure 10 depicts the relationship between heat release rate (HRR) and crank angle (CA) for diesel, E10LHP20, E15LHP30, and E20LHP30. The graph depicts that as ethanol concentration increases, the peak HRR lowers due to the cooling impact and low cylinder temperature, resulting in inadequate combustion. In addition, it was noted that the HRR was greater for conventional fuel operation than for ethanol blend operation. HRR was 67.9 J/°CA for E10LHP20 and 72.34 J/°CA for diesel. Diesel's HRR was greater than E10LHP20, E15LHP30, and E20LHP30 by 4.2%, 11.1%, and 11.9%, respectively. It may be owing to diesel fuel's fast primary combustion, which results in a greater heat release rate. The start of combustion was delayed for ethanol blend combustion than diesel combustion^8,34^. The ethanol blend LHP demonstrated a delayed peak HRR compared to diesel combustion. It could be due to ethanol's lower cetane rating, resulting in a longer ignition delay period and slower burning characteristics. In addition, blends with lower ethanol contents displayed a greater HRR than other blends due to their moderate heating value and nominal combustion temperature. In addition, inferior oxidation rate and delayed burning rate due to suppressing the cylinder's temperature with ethanol are significant factors in the decrease in HRR^26^.

## Impact of LHP and Ethanol blends on SFC, CO, and HC

The creation of a process often employs the use of experimental design to ensure that all aspects of the problem are addressed. This approach makes use of statistical data collected from individual trials in order to foretell the results of a dynamic, complicated, and unpredictable process. The design of experiments (DoE) is a statistical technique used to collect as much information as feasible for this investigation. The main effects and interactions of a study's variables can both be systematically evaluated with a full factorial design. Full and fractional factorial designs with 2 levels and 3 levels, respectively, are the most popular experimental designs employed by researchers. The use of a factorial design would allow the researcher^39^ to examine the interaction between multiple processes or design variables and an outcome^40^. The Minitab 19 software was used for this full factorial design and response optimizer. In this investigation, a full factorial design was adopted for 2 levels and 3 factors with 8 possible combinations. The interaction of engine load, LHP, and ethanol blend has been investigated as the input parameters for the response of fuel consumption, carbon monoxide, and hydrocarbon emissions. Table 6 displays the variables and levels of full factorial design, and the experimental response of a full factorial design is tabulated in Table 7.

**Table 6:** Variables and levels of factorial design.

Variables	Low level	High level
Engine load (kW)	1.1	4.4
Ethanol blend ratio (%)	10	20
LHP blend ratio (%)	20	40

**Table 7:** Experimental output response of LHP and ethanol blends.

StdOrder	RunOrder	Input parameters			Output response		
		Load (kW)	Ethanol (%)	LHP (%)	SFC (kg/(kW h))	CO (vol%)	HC (ppm)
1	1	1.1	10	20	0.636	0.148	54
2	8	4.4	10	20	0.301	0.124	58
3	5	1.1	20	20	0.491	0.141	62
4	4	4.4	20	20	0.391	0.137	66
5	3	1.1	10	40	0.687	0.152	61
6	2	4.4	10	40	0.351	0.137	71
7	7	1.1	20	40	0.731	0.168	67
8	6	4.4	20	40	0.380	0.14	72

### Effect of LHP and ethanol blends on SFC

**Figure 11:**
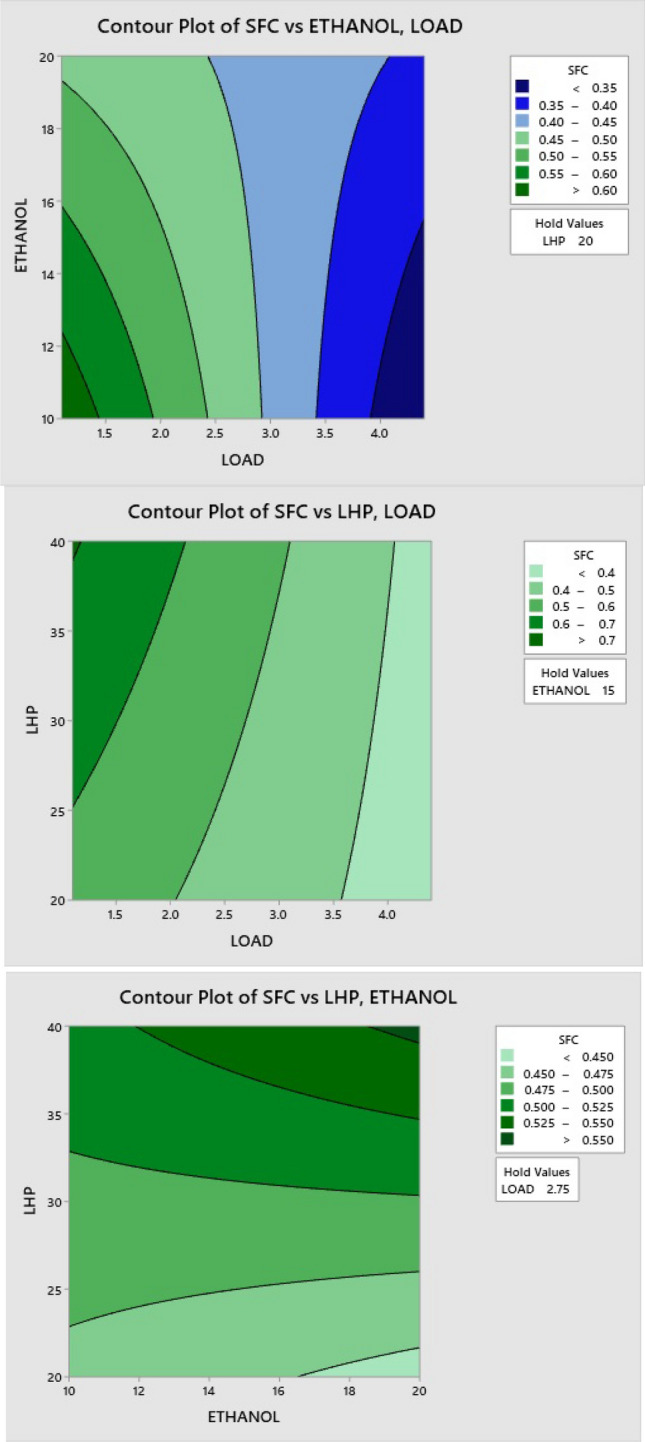
Effect of LHP and ethanol blend ratio on SFC.

The contour plots in Fig. 11 depict the primary effect elements, which include LHP, ethanol blend ratio, and load variation. In these interactions, engine load is beneficial to specific fuel consumption at its maximum, and an increased ethanol ratio results in the lowest SFC. Increased engine power improves fuel combustion by allowing for a more thorough mixing of the fuel and air and improves fuel atomization and cylinder temperature, all of which contribute to efficient combustion and hence reduce specific fuel consumption. Furthermore, due to the viscosity of plastic blends, the SFC increases with an increasing blend ratio, reducing the blend's burning effect at the initial load conditions.

### Effect of LHP and ethanol blends on CO

**Figure 12:**
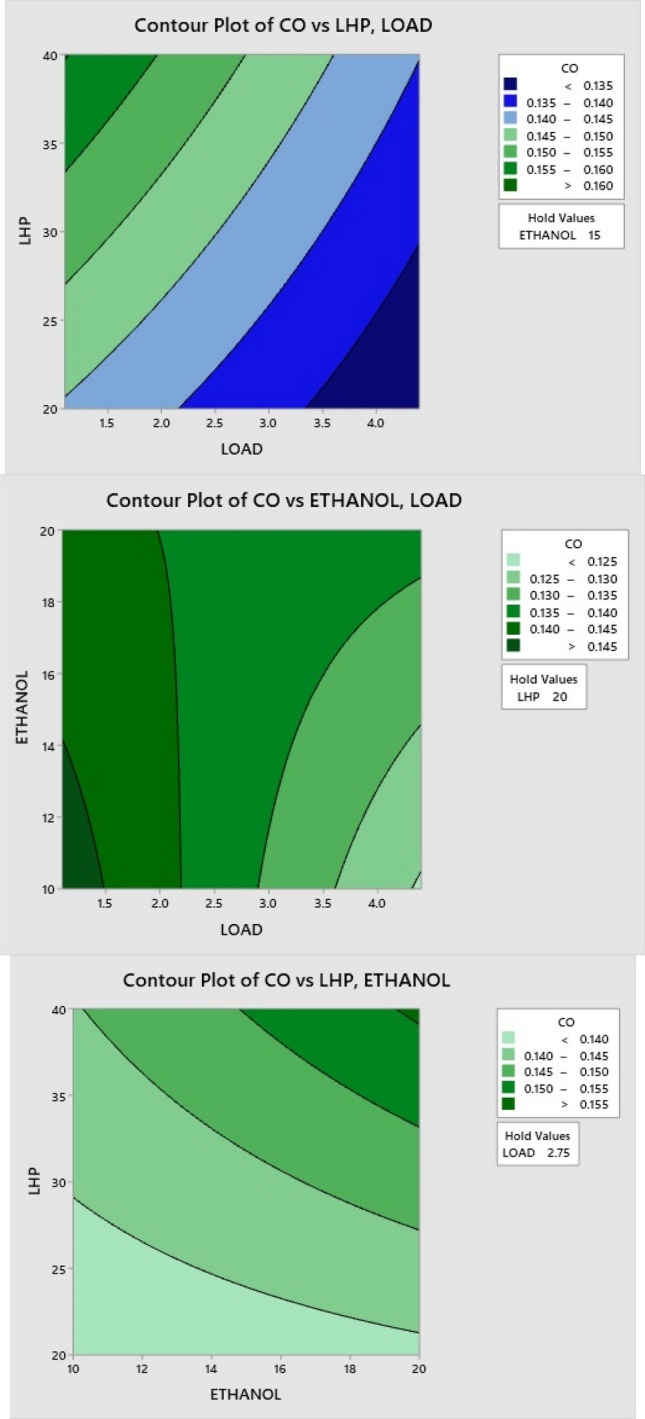
Effect of LHP and ethanol blend ratio on CO.

When the load increases, contour Fig. 12 shows that the carbon monoxide emissions have increased. When the load was low, appropriate combustion resulted in fewer CO emissions; however, when the load was raised, the efficiency of combustion decreased, which resulted in a rise in CO emissions for other LHP and ethanol blends. Blends of ethanol have higher oxygen concentrations, and it promotes complete combustion, which decreases CO concentrations. Incomplete combustion occurs when using LHP blends. Because of their higher content of aromatic components, they burn at a slower rate, and it is harder to break the chain. This leads to higher CO formation at higher ratios.

### Effect of LHP and ethanol blends on HC

**Figure 13:**
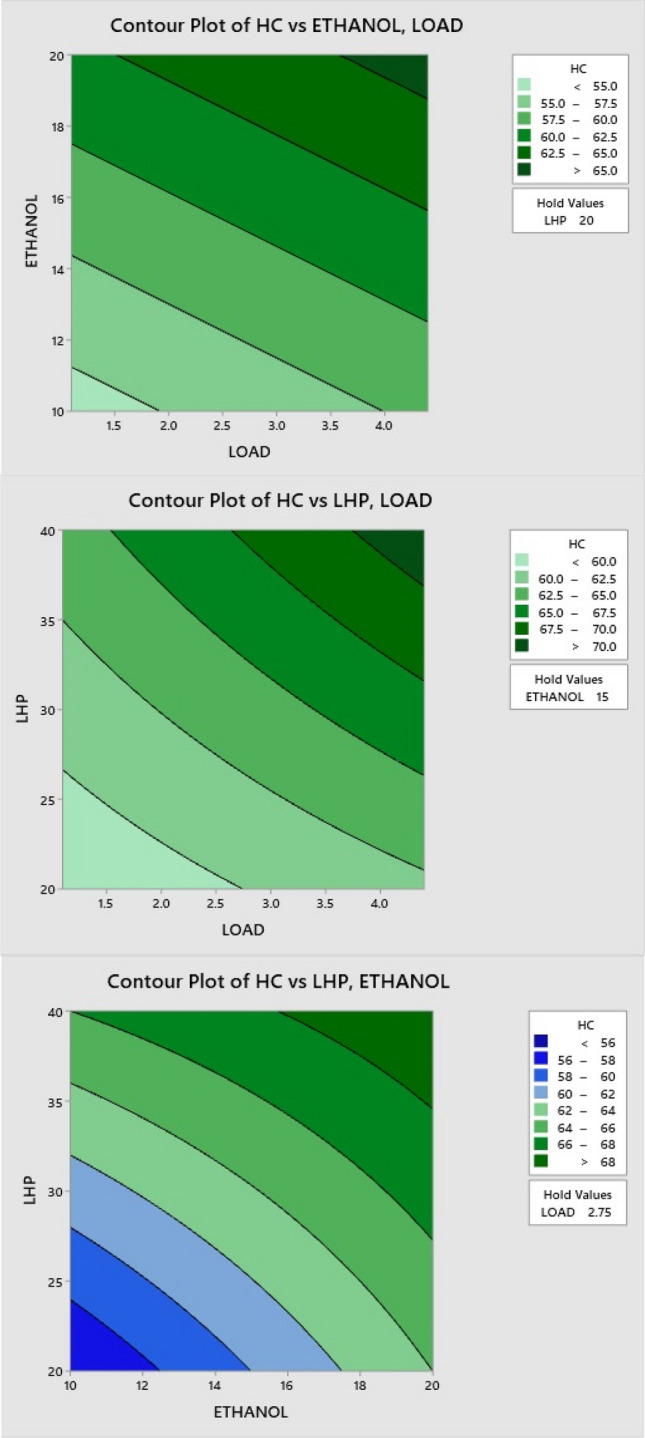
Effect of LHP and ethanol blend ratio on HC.

When the load increases, HC emissions also increase, as shown in the contour Fig. 13. Low vaporization, slow oxidation rates, and high fuel consumption are major contributors to hydrocarbon emission formation. Because of its high oxygen content, ethanol aids in more combustion, reducing HC emissions. Consequently, HC emissions are reduced due to the addition of more oxygen throughout the combustion process on ethanol blend ratios. In contrast, an LHP blend with a larger proportion of aromatic components burns more slowly, and more resistance to breaking the chain is generated, leading to incomplete combustion. At larger ratios, this causes more HC to be produced.

### Response optimization of fuel consumption and emissions

**Figure 14:**
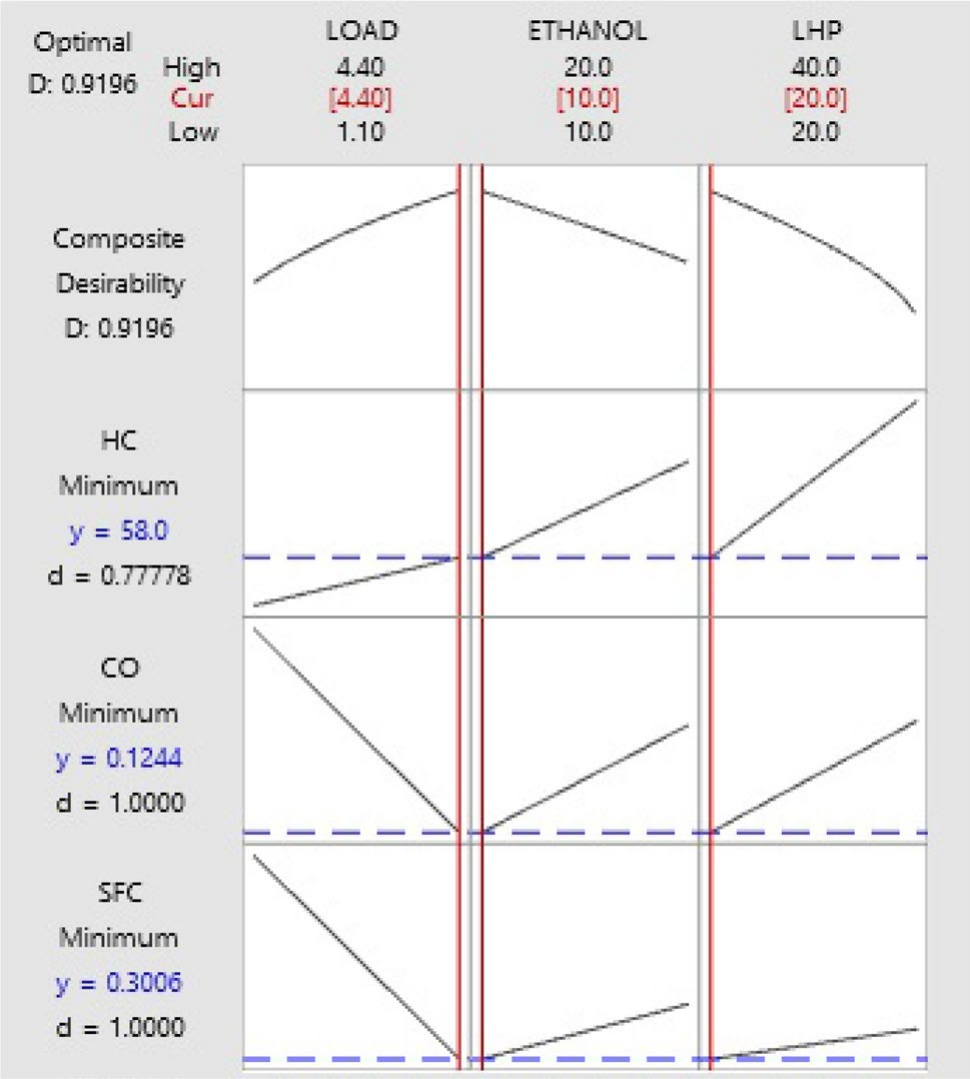
Optimization plot for SFC, CO, and HC.

Get the best possible result from a single response or a group of responses by using Response Optimizer to find the optimal settings for the input variables. Adjusting the input variable values in this interactive visualization can help with sensitivity studies and may lead to a better solution^14^. As illustrated in Fig. 14, the optimization plot demonstrates that the optimal ratio of LHP and ethanol yields the lowest SFC, CO, and HC emissions under maximum load conditions with a composite desirability of 0.9196. Increasing the LHP and ethanol blend ratio will increase all the responses. But the effect on SFC was minimal compared to the effect on CO and HC emissions. Therefore, when decreasing the composite's desirability by minimizing the LHP and ethanol blend ratio, the optimal settings of the LHP and ethanol blend were at the minimum levels in the experiment. This result suggests that researchers should consider experimenting with a lower LHP and ethanol blend ratio for better engine performance. Increasing engine load decreases SFC and CO but causes a minimal increase in HC. The optimal settings for SFC and CO are at the maximum load condition in the experiment. This result implies that experimenting with a higher load may improve engine performance.

The optimal parameters and their response values are listed in Table 8. When using 20% LHP and 10% ethanol, the minimum SFC at maximum load was 0.3006 kg/(kW.h), which is the best possible result. The minimum values of HC and CO emissions were obtained for a ternary fuel blend consisting of 20% LHP and 10% ethanol, and their response is 58 ppm and 0.1244 vol%, respectively, at the full load condition. A confirmation test was used to verify the trial outcomes using observable data, hence validating the response optimizer's output. The fuel consumption is 0.2897 kg/(kW.h), the HC emission is 56 ppm, and the CO emission is 0.1256 vol%, according to confirmation testing results. The results are that 3.6% of the SFC and 1% of the CO, and 3.4% of HC findings deviate from the optimal values determined by full factorial design.

**Table 8:** Response Optimization results of SFC, CO, and HC.

Load (kW)	Ethanol (%)	LHP (%)	SFC (kg/(kW h))	CO (vol%)	HC (ppm)
4.4	10	20	0.3006	0.1244	58

## Conclusion

Waste plastic, a major disposal issue, can be converted into useful energy. The purpose of this study is to explore the effectiveness of plastic fuel in a diesel engine, which is recovered from the waste of low-density polyethylene and high-density polyethylene. Three ratios of LHP and ethanol were used to generate the ternary fuel blends.

- In a summary of the ternary fuel blend investigations, specific fuel consumption was reduced by around 2–7.95% when comparing E10LHP20 to diesel as the base fuel at various load levels. Carbon monoxide emissions were reduced by 3.4–10.2% when compared with the base diesel for E10LHP20.
- Under varying loading conditions, the hydrocarbon content of ternary blend E10LHP20 is reduced by around 6–13.43% compared to diesel fuel. Because the ternary blend contains ethanol that is high in oxygen content, thus the combustion process was improved, which leads to a decrease in emissions.
- E10LHP20, E15LHP30, and E20LHP40 blends have been recorded as having a maximum of 12.4%, 22%, and 46% more nitrogen oxides than base diesel at various load conditions. This is because ethanol produces more NOx emissions due to its higher oxygen content which is available for burning.
- LHP has a higher content of aromatic components; they burn at a slower rate, and it is harder to break the chain. This leads to more smoke emissions due to the higher concentration of ternary blends.
- The minimum values of SFC, CO, and HC emissions were obtained for a ternary fuel blend consisting of 20% LHP and 10% ethanol at the full load condition, and their better results obtained were 0.3006 kg/(kW h), 0.1244 vol%, and 58 ppm, respectively.

On summary, the hydrocarbons found in used plastic represent a valuable alternative source of energy. The most difficult aspect of waste plastic recycling is the possibility of converting the plastic into usable energy. The findings indicate that it might replace fossil fuels in a variety of applications, including diesel locomotives, industrial boilers, and even marine propulsion systems.

## Data Availability

The datasets used and analyzed during the current study are available from the corresponding author on request.

## Abbreviations

ANOVA: Analysis of variance
BTE: Brake thermal efficiency
bTDC: Before top dead centre
C: Carbon
CA: Crank angle
CO: Carbon monoxide
DOE: Design of experiments
E: Ethanol
E10LHP20: 70% Diesel + 20% LHP + 10% Ethanol
E15LHP30: 55% Diesel + 30% LHP + 15% Ethanol
E20LHP40: 40% Diesel + 40% LHP + 20% Ethanol
FFD: Full factorial design
GC–MS: Gas chromatography–mass spectrometry
H: Hydrogen
HC: Hydrocarbons
HDPE: High-density polyethylene
HRR: Heat release rate
IP: In-cylinder pressure
kW: Kilo Watts
LDPE: Low-density polyethylene
LHP: Low and high density plastic pyrolysis oil
NOx: Nitrogen oxides
O: Oxygen
PE: Polyethylene
PET: Polyethylene terephthalate
PPO or PO: Plastic pyrolysis oil
PP: Polypropylene
ppm: Parts per million
PS: Polystyrene
SFC: Specific fuel consumption
W: Waste plastic oil
WPO: Waste plastic oil
ZSM-5: Zeolite socony mobil-5
